# Mid-range visual deficits after stroke: Prevalence and co-occurrence

**DOI:** 10.1371/journal.pone.0262886

**Published:** 2022-04-01

**Authors:** Nikki A. Lammers, Nils S. Van den Berg, Selma Lugtmeijer, Anouk R. Smits, Yair Pinto, Edward H. F. de Haan, the visual brain group

**Affiliations:** 1 Department of Psychology, University of Amsterdam, Amsterdam, The Netherlands; 2 Department of Neurology, University Medical Center Amsterdam, Amsterdam, The Netherlands; 3 Department of Neurology, University Medical Center Groningen, Groningen, The Netherlands; 4 Donders Institute for Brain, Cognition and Behavior, Radboud University, Nijmegen, The Netherlands; 5 Department of Neurology, University Medical Center Utrecht, Utrecht, The Netherlands; 6 St.Hugh’s College, Oxford, United Kindom; University of Canberra, AUSTRALIA

## Abstract

Visual deficits are common after stroke and are powerful predictors for the chronic functional outcome. However, while basic visual field and recognition deficits are relatively easy to assess with standardized methods, selective deficits in visual primitives, such as shape or motion, are harder to identify, as they often require a symmetrical bilateral posterior lesion in order to provoke full field deficits. Therefore, we do not know how often they occur. Nevertheless, they can have severe repercussions for daily-life functioning. We aimed to investigate the prevalence and co-occurrence of hemifield “mid-range” visual deficits (i.e. color, shape, location, orientation, correlated motion, contrast, texture and glossiness), using a novel experimental set-up with a gaze-contingent presentation of the stimuli. To this end, a prospective cohort of 220 ischemic (sub)cortical stroke patients and a healthy control group was assessed with this set-up. When comparing performance of patients with controls, the results showed that deficits in motion-perception were most prevalent (26%), followed by color (22%), texture (22%), location (21%), orientation (18%), contrast (14%), shape (14%) and glossiness (13%). 63% of the stroke patients showed one or more mid-range visual deficits. Overlap of deficits was small; they mostly occurred in isolation or co-occurred with only one or two other deficits. To conclude, it was found that deficits in “mid-range” visual functions were very prevalent. These deficits are likely to affect the chronic post-stroke condition. Since we found no strong patterns of co-occurrences, we suggest that an assessment of deficits at this level of visual processing requires screening the full range of visual functions.

## Introduction

Visual deficits are frequent complications after stroke [1–5]. These deficits can have a significant negative effect on the patients’ long-term outcome, as assessed with the Barthel or the Frenchay checklist [6], and quality of life [7]. They can have a chronic impact on general mobility, the ability to judge distances or motion speed and comes at high economic costs [8–10].

Visual deficits after stroke can vary from visual field defects, deficits in perceiving basic visual features to higher-order recognition deficits (e.g. recognizing faces). Hemianopia or scotoma’s can be measured easily, and are well documented in the literature [7]. In addition, the repercussions of these defects for daily living are well understood [11]. Similarly, recognition deficits, such as object agnosia and prosopagnosia, are relatively easy to assess and have been studied extensively [12, 13].

However, there is an intermediate stage in the cortical processing of visual information that has remained largely uncovered in clinical practice. The classic cases of selective deficits in ‘mid-range’ visual functions, such as motion [14, 15] or shape [16] are rare because they require symmetrical bilateral posterior lesions in order to provoke full field deficits. The physiology of this intermediate stage of visual processing is well documented. More than forty retinotopic maps have been discerned [17–19], populated by neurons tuned to different aspects of the visual world, such as orientation, shape and motion [20]. Unilateral damage to the posterior brain may result in selective deficits in these visual cues or may affect only the contralesional visual hemifield. These latter deficits can be construed as partial hemianopic impairments affecting only one visual dimension.

The assessment of these visual hemifield defects is hampered by the fact that most available tests only measure a couple of visual dimensions (e.g. the Farnsworth Munsell test for color perception or the Benton Line-orientation test for perceptual organization and spatial perception). The Leuven Perceptual Organization Screening Test (L-POST) [21, 22] is an excellent test measuring a wide range of mid-level visual perceptual functions, but is, like most screening tasks a free viewing task that allows the subject to make head and eye movements. Therefore, the patient can easily compensate for hemifield defects by moving stimuli into the spared hemifield. For example, Short and Graff-Radford (2001) [23] described a patient with hemiachromatopsia, who could not identify the colors that were demonstrated in the right visual field. However, the color perception deficit could not be demonstrated with a free viewing task. Note that this head- and eye movement compensation does not preclude the possibility that these deficits can affect the patient in daily living. This possibility is supported by observations that stroke patients often complain about “diffuse” visual problems [24] and are at higher risk for falling accidents [25]. For instance, although the patient described by Short and Graff-Radford (2001) [23] was not aware of her deficit, when asked about visual problems, she acknowledged something was wrong with her vision but she did not know what it was. In addition, a recent study showed that 40% of the patients with post stroke visual impairments cannot adequately report these visual problems, although these impairments could affect daily functioning [26].

At present, it is unknown how often these mid-range visual deficits occur and whether they occur in isolation or whether there is a trend for some functions to cluster together. The current study was devised to establish the prevalence and clinical characteristics of deficits in eight important ‘mid-range’ visual functions, and evaluate their co-occurrence in a large prospective cohort of stroke patients. To test the visual functions, we used a novel experimental set-up with a gaze-contingent presentation of the test stimuli.

## Materials and methods

### Study setting

This study is part of a prospective, multi-center cohort study ‘A Functional Architecture of the Brain for Vision’ (FAB4V). The objective of FAB4V is two-folded:1) to examine the functional architecture of the visual brain based on necessity and the theoretical framework of cortical networks and 2) to investigate the frequency and severity of visual and cognitive impairments following ischemic stroke. The 220 patients that were included in the study were admitted to one of the following hospitals in the Netherlands: Amsterdam University Medical Center (Amsterdam UMC), Radboud University Medical Center (Radboudumc), University Medical Center Groningen (UMCG), University Medical Center Utrecht (UMCU), Onze Lieve Vrouwe Gasthuis (OLVG), Maasziekenhuis Pantein, Rijnstate, Ommelander Ziekenhuis Groep, St. Antonius Ziekenhuis and Diaconessenhuis. Data collection took place from September 2015 until January 2020. Assessment took place at one of the four academic hospitals. The study was approved by the Medical Ethics Committee Utrecht (METC-nr 2015.372). All participants signed a written informed consent prior to their participation and were treated in accordance with the Declaration of Helsinki.

### Participants

A group of 220 patients (age 18–90 years) with a diagnosis of ischemic stroke in (sub)cortical areas distributed over the entire supratentorial region were included in the study. Diagnosis was made by an expert neurologist based on the presence of an acute focal deficit. Lesion-presence was confirmed by MRI or CT.

A reference group was used to determine the thresholds (fixed values) of the task based on an adaptive procedure. This reference group included 62 age-matched healthy controls. Subsequently, a second age-matched Healthy Control (HC) group (n = 49) was included. In contrast to the reference group, who performed the adaptive task, the HC group could directly be compared with the patient group as both these latter groups performed the fixed value task.

HCs were volunteers who were recruited by word or mouth or via the Senior Lab of the University of Amsterdam (www.seniorlab.nl). Exclusion criteria for both patients and controls consisted of serious neurological disorders (other than ischemic stroke in case of patients), psychiatric disorders, substance abuse, or insufficient command of the Dutch language.

### Visual assessment

Prior to all tasks, patients were assessed with a screening for visual field deficits (see S1 File). Patients with a complete homonymous hemianopia were not included in the analyses.

Eight experimental tasks were constructed for the assessment of perception-proficiency of *color* (isoluminant stimuli in the red-green range), *shape* (Efron shapes), *location* (dot in a circle), *orientation* (lines at different angles), *contrast* (bars with converging grey-level differences), *glossiness* (surface differences in shining), *texture* (from Brodatz grayscale texture album) [27] and *correlated* motion (different percentages of dots moving in the same direction).

**Fig 1:**
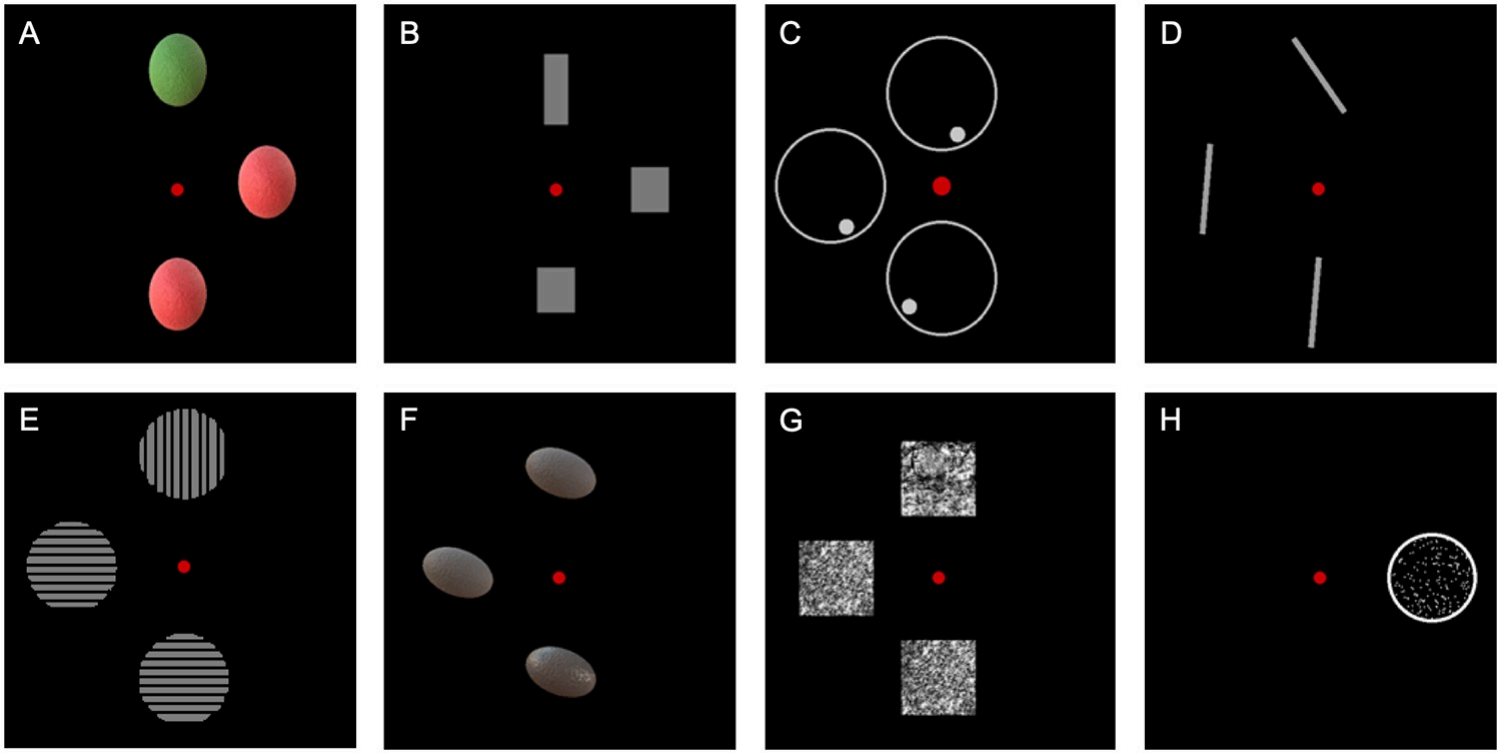
Examples of stimulus-pictures used to assess the perception of A) color, B) shape, C) location, D) orientation, E) contrast, F) glossiness, G) texture and H) correlated motion.

These eight visual functions were chosen as these are commonly assessed visual functions in vision research [28, 29]. With regard to the color perception task, the red-green range was chosen based on previous studies assessing color perception [28, 29]. All participants were asked whether they had difficulties with color perception, to exclude congenital color deficiencies. Examples of the stimuli are presented in Fig 1 (see S1 File for a detailed description).

**Fig 2:**
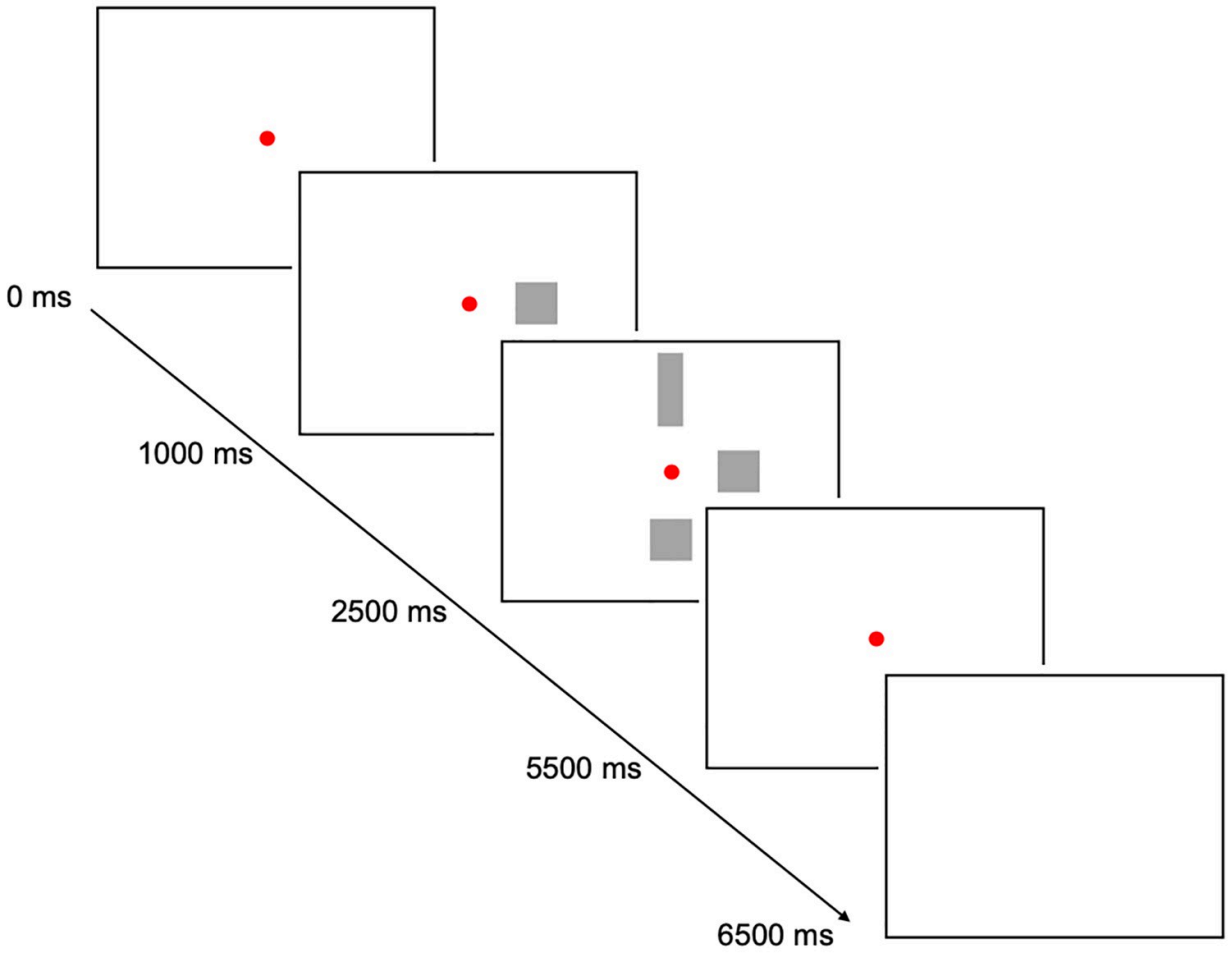
Schematic overview of the task-paradigm for the assessment of shape-perception with the cumulative time in ms.

For all tasks, participants were positioned behind a 21.5-inch computer screen (Benq GL2240, resolution 1920 x 1080) with their chin on a chinrest. The viewing distance was 66 centimeters. All tasks, except for correlated motion, employed a similar paradigm in which each trial started with a red fixation dot at the center of a black screen (see Fig 2 for a schematic overview of an example of the task-paradigm). This fixation dot remained visible for the entire trial. The subject was instructed to keep his/her gaze fixed on this dot throughout the trial. The target stimulus appeared one second after the start of the trials and remained visible for 1.5 seconds, without the presence of the response items. The target was presented on the horizontal midline, at 5° to the right or the left side of the fixation dot. Subsequently, two response items appeared for 3 seconds beside the target item on the screen. One response item appeared 5° below and one at 5° above the red fixation dot. One of the response items was identical to the target and the other response item differed from the target stimulus in a systematic fashion. The patient had to indicate which of the two response items was identical to the target stimulus within four seconds after the response items appeared. The first three seconds the response items were visible on the screen, and in the last second only a blank screen with a fixation dot was presented. If the response was not within the four seconds after the stimuli were presented, the trial was coded as incorrect. The task assessing motion perception only involved a target stimulus with moving dots in it. The percentage coherence of these moving dots was adaptively changed across trials and participants had to indicate whether the dots were moving upwards or downwards. All tasks started with twelve practice trials (six per hemisphere), followed by 24 assessment trials (twelve per hemifield in a randomized manner). The presentation was blocked per task. Responses could be given by the use of a joystick or could be given verbally. This made the task suitable for aphasic patients or patients with motoric difficulties.

An eye-tracker (Eyelink 1000; SR Research Ltd, Canada) was used to register and control for eye fixations and movements. The target presentation was controlled in a gaze-contingent manner, so that the target always remained in the same retinal position (of the horizontal axis), independent of eye movements, allowing for separate hemifield-testing. For example, if participants made eye-movements to the left, the stimulus shifted along to the left. If participants moved their eyes so far to one side of the screen that the target ‘fell off’ the screen, the trial was aborted and replaced with another trial.

The twelve practice trials before the start of each task were meant to practice the testing procedure and to test whether participants were able to inhibit eye saccades. When participants could not inhibit eye saccades (in which case the response items kept moving across the screen from left to right), participants were allowed to perform another series of practice trials. If participants could then still not inhibit eye saccades or when tracking eye-movements was hampered by e.g. drooping eyelids, a ‘short-presentation’-procedure without eye-tracker was performed. In this procedure, target stimuli were presented for 200 milliseconds. The response pictures remained visible for the entire trial of 5.5 seconds. Subjects had to respond within these 5.5 seconds. This short presentation-time prevented patients from moving their eyes to the left or right side, so that separate hemifield-testing remained possible. The stimuli and procedure for this ‘short-presentation’-mode were similar to the stimuli and procedure of the gaze-contingent presentation of the stimuli.

### Determination of present difference values

First, the reference group performed a series of tasks with an adaptive staircase procedure. In these *adaptive* tasks, an exact threshold per hemifield for each task was determined through an adaptive staircase procedure, i.e. the difference value between the target stimulus and the odd response item was adaptively changed. The mean threshold of the reference group on these *adaptive* tasks plus 1.64 standard deviations (SD) was used as the present fixed difference value for the visual tasks. Both the patient group and the HC group performed these visual tasks with the fixed difference value. The score was the correct number of responses with a maximum score of 12 per hemifield. The mean score minus 1.64 SDs of the HC group on the fixed visual tasks were used as the cut-off score for patients; a score of patients below this cut-off was treated as deviant. Only for the motion-task, a different procedure was used. This task consisted of 48 trials (24 trials per hemifield) in which the threshold per hemifield was determined through an adaptive procedure. The total testing time took approximately sixty minutes. For the analyses of all tasks except motion perception, the lowest score (L or R) of the patients (possible range 0–12) was compared with the cutoff of the corresponding HC group (mean– 1.64 * SD of either the left or right hemifield). For motion perception, the highest score (L or R) of the patients was compared with the cutoff of the corresponding HC group (mean + 1.64 * SD of either the left or right hemifield), because this task had an adaptive procedure in which lower scores indicated better performance.

## Results

### Participants

#### Reference sample

Sixty-two age-matched controls (mean age = 57.6, *SD* = 14.5) were included in the reference group to determine the pre-set fixed values for the visual sensory tasks. Thirty-eight people of this reference group performed the tasks with the gaze-contingent presentation of the stimuli and 24 performed the task with a ‘short presentation’-procedure of the stimuli. The 62 controls of the reference group were randomly assigned to the gaze-contingent condition or the short presentation condition. In that way, we were able to establish norms for both conditions. Scores on the visual tasks did not significantly differ between the short condition and the gaze-contingent condition (all *p*s >.05).

#### Patients and healthy controls

The clinical characteristics of the patients are presented in Table 1. In addition, a group of 49 HCs were included (mean age = 59.7, *SD* = 13.3). There were no significant differences between patients and the HC group regarding age (*t* = 1.13, *p* = .263) and sex (*χ*^*2*^ = .69, *p* = .407). However, the educational level in the HC group was significantly higher (*t* = 4.19, *p* = .000).

**Table 1:** Clinical characteristics of the patient group.

Characteristic	Patients (*n* = 220)
Age (M ± SD)	59.7 ± 13.3
Sex, %men	144, 65.5%
Education level (M ± SD)	5.2 ± 1.3
NIHSS (M ± SD) *	6.6 ± 6.3
Time since stroke (in weeks) (M ± SD)	7.8 ± 3.8
Left hemispheric lesion (*n*)	93
Right hemispheric lesion (*n*)	90
Bilateral lesion (*n*)	37

*Note*. Education (Verhage, 1964): seven-point scale, on a range from 1 (primary education) to 7 (university education); NIHSS = National Institutes of Health Stroke Scale.

Available for 135 of the 220 patients.

### Visual functions

**Fig 3:**
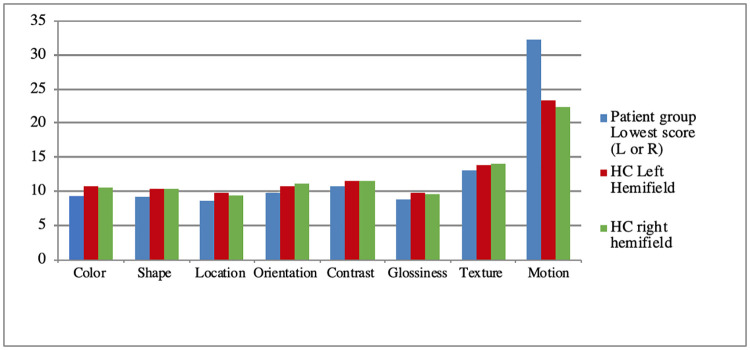
The mean scores for the patient group and the HC-group. For motion perception, lower scores indicate better performance. For all other tasks, higher scores indicate better performance.

Most patients were able to fixate. In only six percent of the patients, we were not able to successfully track eye-movements during the practice trials. In these instances, we used the short-presentation of the stimuli (without eye-tracker). When patients did not perform all tasks (because of e.g. fatigue or time shortage), we registered this as missing data for those particular visual features. Table 3 shows the number of patients by which a particular task has been performed. In Table 2, the means, standard deviations and range of test scores of the patients (the lowest score on either the left or the right hemifield (possible range 0–12)) and the HC-group scores are presented, separated for the left and the right hemifield. This information is also shown in Fig 3. Note that higher scores indicate better performance, except for motion perception; the motion perception task is an adaptive task where lower scores indicate better performance. Table 3 shows the percentages of patients with deficits per visual feature for the entire patient group. A score was treated as impaired when the threshold was in the lowest 5% of the HC group (M—1.64*SD). As can be seen, the percentages of deficits ranged from approximately 13–14% (glossiness, shape and contrast) up to 26% (motion).

**Table 2:** Means (M), Standard deviations (SD’s) and ranges of scores of the patient group and the HC-group.

	Patient group	HC-group	
	Lowest score (L or R)	Left hemifield	Right hemifield
	M (SD), Range	M (SD), Range	M (SD), Range
Color	9.4 (1.9), 2–12	10.6 (1.2), 8–12	10.5 (1.4), 7–12
Shape	9.2 (1.8), 4–12	10.2 (1.7), 5–12	10.2 (1.5), 7–12
Location	8.6 (2.2), 2–12	9.7 (9.4), 5–12	9.4 (1.7), 5–12
Orientation	9.7 (2.0), 2–12	10.6 (1.7), 5–12	11.0 (1.4), 6–12
Contrast	10.7 (1.7), 4–12	11.4 (1.4), 6–12	11.4 (1.2), 6–12
Glossiness	8.7 (2.8), 2–12	9.7 (2.3), 3–12	9.6 (2.3), 5–12
Texture	13.0 (2.1), 6–12	13.7 (1.5), 10–16	14.0 (1.5), 11–16
Motion	32.2 (15.5), 12–100	23.3 (9.3), 16–56	22.4 (7.4), 16–48

**Table 3:** Frequencies of mid-range visual deficits (in percentages).

Mid-range Feature	*n*	% impaired
Color	175	22%
Shape	213	14%
Location	204	21%
Orientation	200	17%
Contrast	177	14%
Glossiness	156	13%
Texture	176	22%
Motion	131	26%

**Fig 4:**
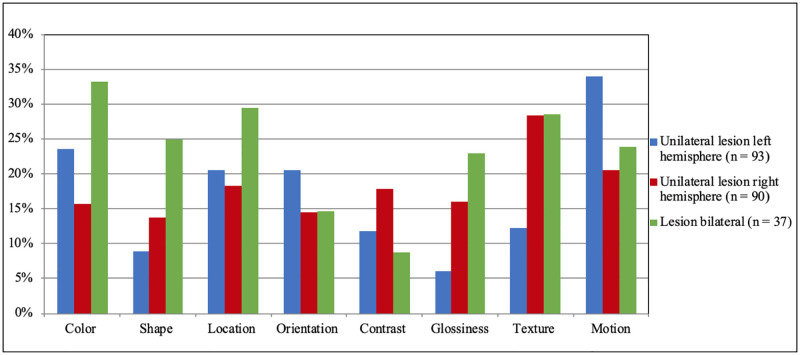
Frequencies of mid-range visual deficits after stroke, split for lesion lateralization.

In Fig 4, percentages of deficits are shown separately for the lesion-laterality subgroups (i.e. left, right or bilateral lesions). Overall, deficits occurred in all laterality-subgroups. Chi-square tests also showed no significant differences between the lesion-laterality subgroups on most visual features. Only texture-perception deficits were significantly less prevalent after left-hemispheric stroke compared to right and bilateral hemispheric stroke (*χ*^*2*^ = 6.71, *p* = .035). Correlations between mid-range functions and lesion volume were low and only significant for orientation (*tau* = -.150, *p* = .007) and motion (*tau* = -.182, *p* = .008). Correlations between mid-range functions and the NIHSS-score were low (all *r*s < .20) and non-significant (all *p*s > .05), except for glossiness (tau = .16, *p* = .041).

### Clusters of visual deficits

Table 4 shows the co-occurrence of two visual deficits (as percentages of all participants who performed both tasks). Overall, overlap is small and deficits tend to occur in isolation or, sometimes, co-occur with only one or two other deficits (Table 5). Across the board, impairment affecting all or most visual functions does not happen.

**Table 4:** Percentages of co-occurrence of specific visual deficits. How many patients who have performed both task x and y (in %) have deficits in both tasks.

x/y	Color	Shape	Location	Orientation	Contrast	Glossiness	Texture
Shape	6.9%						
Location	6.0%	4.9%					
Orientation	3.7%	5.0%	6.2%				
Contrast	3.4%	3.4%	5.1%	5.8%			
Glossiness	1.6%	3.2%	5.2%	2.0%	1.4%		
Texture	3.6%	3.6%	7.1%	6.0%	3.1%	8.7%	
Motion	6.3%	3.9%	3.9%	7.0%	2.5%	1.0%	6.3%

**Table 5:** Frequency-table: On how many tasks do patients show deficits?

Number of visual tasks on which a patient shows deficits	Frequency, *n (% of total n*)
0	81 (36.8%)
1	69 (31.4%)
2	41 (18.6%)
3	19 (8.6%)
4	4 (1.8%)
5	5 (2.3%)
6	1 (0.5%)
7	0 (0%)

## Discussion

In this study, we investigated the prevalence of deficits in mid-range visual functions after stroke, and whether these deficits tend to co-occur in specific clusters. To this end, we devised a novel experimental set-up which allowed us to test the perception of color, shape, location, orientation, contrast, texture and motion restricted to one hemi-field using a gaze-contingent presentation procedure.

The first finding is that deficits in these mid-range visual functions are very prevalent, regardless of lesion-laterality or lesion size. In fact, 63% of the stroke patients showed one or more mid-range visual deficits. Highest frequencies of deficits were found in the perception of motion (26%) and color (22%). The frequency of the other visual deficits varied between about 13% and 22%. The least prevalent deficits concerned the perception of glossiness, shape and contrast (about 14%), which could be the result of the fact that there are only a handful of patients with damage to the primary visual cortex. The high prevalence of visual hemifield deficits is not surprising, as more than a quarter of the human cortex is involved in the processing of visual information [30]. This finding may have important clinical implications. In a broad sense, we know that visual deficits can be powerful predictors of the chronic functional (e.g. Barthel screening) and neuropsychological outcome [1, 6]. However, those findings were based on standard neuropsychological tests, e.g. Rey-Osterrieth Complex Figure-copy and Judgment of Line Orientation. With respect to mid-range visual deficits, we are in uncharted territory. At present, it is unclear what the ramifications are for functional outcome and daily-living. For example, we can now only guess to what degree a selective motion-perception deficit might affect perceiving oncoming traffic while driving. As mentioned earlier, we do know that stroke patients often complain about visual problems [24] and that they are at higher risk for falling accidents [25]. Moreover, previous case-studies have shown that selective deficits in mid-range visual functions can have knock-on effects on higher-order visual disorders, affecting daily-life functioning [29, 31].

The second finding concerns the observation that overlap of these mid-range deficits is small, and that they tend to occur in isolation or co-occur with only one or two other deficits. Therefore, the assessment of deficits at this level of visual processing requires screening a broad range of visual functions. For example, one cannot assume that there are no deficits in this realm if one has excluded a motion perception problem. The relative independence of the mid-range visual processing was, however, to be expected on the basis of the physiology of the visual system where many different retinotopic maps with different functional characteristics have been described [17–19]. The lack of clustering is in line with models, such as the “patchwork” model proposed by de Haan and Cowey [30], which postulate that there are overlapping visual networks with many interconnections, which are widespread throughout the brain.

A more practical observation is that the majority of patients were able to perform most of the tasks. Some patients needed some practice and the requirement to maintain fixation was seen as taxing. Nevertheless, the procedure seemed feasible. Trials in which patients were unable to maintain fixation were discarded and immediately repeated. Only when patients repeatedly showed extensive eye movements on the majority of the tasks, they were left out the analyses as in that case low scores could be a result of a fixating problem instead of a deficit in visual processing. Moreover, the fact that responses could be given either verbally or by using a joystick makes the task-procedure also suitable for patients with motoric difficulties or aphasic patients. Therefore, it could be of interest to test the clinical applicability of this experimental set-up in future studies. This could be a valuable addition to other computerized visual tasks, such as The Leuven Perceptual Organization Screening Test (L-POST) [21, 22].

This study is subject to some limitations. The first concerns the current statistical methodology, as the data are not normally distributed. The current approach could lead to an overestimation of the deficits. It should also be noted that some controls also performed below the cutoff. The second limitation is the missing data for some visual tasks. Because of fatigue or time shortage, the visual assessment could not always be completed. However, all tasks have still been performed by a large number of patients. Third, the age of the patient group is younger than the average stroke patient. We wanted to exclude comorbidities as much as possible. The older the patient, the larger the chance of comorbidities. This would make it more difficult to state whether there would be a specific visual problem or a more general decline in cognitive functioning. The relatively younger group of patients could on the one hand limit the generalisability of the data to all stroke patients with regard to the percentage of patients having a deficit. On the other hand, it would be expected that the inclusion of older patients would have led to an even higher percentage of impaired visual functions, which even more highlights the merit of assessing visual deficits. In addition, the educational level of the HC group was higher than the patient group. However, we do not expect this to influence the results, as, although slightly higher in HC’s, the mean educational level of both the HC group and the patient group was in-between intermediate and higher vocational education (between 5 and 6 on the Verhage-scale). Another limitation of our study is the use of a fixed value rather than using an adaptive task. An adaptive task would have been preferable as this gives more detailed information, such as whether a patient’s threshold co-varies with other aspects of performance. However, it became apparent that threshold measurements would be too time consuming for our patient population. When considering a translation of our experimental setup into a diagnostic tool for clinical practice, it would be helpful to reduce the testing time. Furthermore, it could be helpful to simplify the user interface in order to facilitate the ease of use for clinicians. This could also help in reducing the testing time.

In sum, we have demonstrated that various mid-range visual deficits were very prevalent after stroke, regardless of lesion laterality or lesion size. In addition, we found that these mid-range deficits mainly occurred in isolation or co-occurred with only one or two other deficits. We have developed a novel experimental set-up for visual assessment that may help assessing these deficits in stroke patients. The high prevalence of visual deficits after stroke implies that the measurement of these deficits could be of clinical significance. However, a detailed description of the repercussions of the different deficits needs to be discerned in future studies.

## Supporting information

S1 File Detailed description of the visual assessment. (DOCX)

S1 Dataset (CSV)
