# Supplementary material for: Mid-range visual deficits after stroke: Prevalence and co-occurrence
Source: PLoS One. 2022 Apr 1;17(4):e0262886. doi: 10.1371/journal.pone.0262886 (PMC8975013; doi:10.1371/journal.pone.0262886)
Supplement: S1 File — (DOCX) [file pone.0262886.s001.docx]

**S1. Supplementary materials**

**Detailed description of the visual assessment**

**Visual field screening.** In this screening, subjects were asked to focus on a red fixation dot at the center of a black screen (xyY =0, 0, 0 cd/m^2^). The visual field had a width of 49.8 degrees and a height of 27.6 degrees. The fixation dot remained visible for the entire trial. Then, in total, 45 stimuli (grey dots) (xyY = 0.286, 0.314, 30.4 cd/m^2^ ) with a diameter of 0.509º of visual angle were presented, one at the time, at each quadrant of the visual field and at the center of the screen (9 per quadrant) for 100 milliseconds per stimulus. The quadrants included the upper left corner (0, 0, xmid, ymid); upper right (xmid, 0, X, ymid); below left (0, ymid, xmid, Y); below right (xmid, ymid, X, Y); center (0.5*xmid, 0.5*ymid, 1.5*xmid, 1.5*ymid). The location within the quadrant was chosen randomly, but at least a distance of the radius of the dots (0.255º of visual angle) from all borders (top, bottom, left and right). Subjects were instructed to indicate when they perceived a stimulus-dot. The response time was 1200 milliseconds. The time between the stimuli differed across trials (a range between 100ms and 300 ms; the time within this range was chosen randomly). The location of the stimulus was chosen randomly. The eye tracker was used to control for eye-movements; when the subject made eye-movements during the presentation of a stimulus, the respective stimulus was replaced with another trial at a later stage of the task. A score below 5 out of 9 was treated as deviant. Patients with a complete homonymous hemianopia (deviant performance in two quadrants in the same hemifield) were excluded from analyses.

**Color perception.** The target stimulus and response items for the assessment of color discrimination were picked out of 51 isoluminant colored circles with a diameter of 3.5º of visual angle. These colored circles ranged in equal steps on the Commission Internationale de l'Eclairage (CIE) chromaticity spectrum between green (xyY = 0.286, 0.467, 14.986 cd/m^2^) and red (xyY = 0.450, 0.322, 8.854 cd/m^2^). Subjects were asked to indicate which of the response items was identical to the target stimulus. The *adaptive* task started with a difference value between the target stimulus and the odd response item of 23 color steps for both hemifields. The target item could have any value between 1 and 51. The difference values were adaptively changed, i.e. after three right or three wrong responses, the difference value became smaller or larger respectively, with an initial step size of 4 steps. When a difference value of 11 steps had been reached, the step size was reduced to 1 step.

The fixed difference value between the target stimulus and the odd response item used for the fixed visual task (i.e. the mean threshold of the reference group + 1.64**SD*) was 11 color steps. The cut-off score for the fixed visual task (mean score of the HC group on this fixed visual task minus 1.64*SD was 8.7 (left visual field) or 8.2 (right visual field). A score of patients on this task below this cut-off was treated as deviant.

**Shape perception.** The target stimulus and response items for the assessment of shape perception were selected from 60 grey shapes (xyY = 0.283, 0.309, 13.928 cd/m^2^), gradually changing from a rectangle (shape one; length 7.8 º and width: 0.5º of visual angle) to a square (shape 60; size: 2º x 2º of visual angle). These stimuli differed in length and width, but always had the same area. The adaptive task started with a difference value between the target stimulus and the odd response item of 26 shape-steps for both hemifields. The target stimulus could have any value between 1 and 60. After three right or three wrong responses, the difference value became smaller or larger respectively, with an initial step size of 4. This way, the shape of the square changed in such a way that it resembled the rectangles more and vice versa. When a difference value of 14 shape-steps had been reached, the step size was reduced to 1 step. The mean threshold of the reference group + 1.64**SD,* used as the fixed difference value for the fixed visual task, was 14 shape-steps. The cut-off score for the fixed visual task (mean score of the HC group on this task minus 1.64*SD was 7.4 (left visual field) or 7.8 (right visual field). A score of patients below this cut-off was treated as deviant.

**Location perception.** Circles with a dot in it were used for the assessment of location perception. The circle (xyY = 0.285, 0.312, 23.766 cd/m^2^) had a diameter of 3.5º of visual angle and the dot (xyY = 0.285, 0.312, 23.766 cd/m^2^) had a diameter of 0.6 º of visual angle. This dot was located at 0.1º of visual angle of the edge of the circle. For the adaptive task, the difference between the location of the dot in the circle of the target stimulus versus the location of the dot in the response item was adaptively manipulated and was initially set to 46 degrees for both hemifields. After three right or three wrong responses, the difference value became smaller or larger respectively, with an initial step size of 8 degrees. When a difference value of 22 degrees between the target stimulus and the response item had been reached, the step size was reduced to 2 degrees. The fixed difference value for the fixed visual task (i.e. the mean threshold of the reference group + 1.64**SD*) was 22 degrees. The cut-off score for the fixed visual task (mean score of the HC group on this task minus 1.64*SD was 6.3 (left visual field) or 6.5 (right visual field). A score of patients below this cut-off was treated as deviant.

**Orientation perception.** The target stimulus and response items for the assessment of orientation perception involved straight lines (xyY = 0.285, 0.312, 23.766 cd/m^2^, length = 4º of visual angle) with a varying orientation. The odd response item had a different orientation than the target stimulus. For the adaptive task, the difference in orientation between the target stimulus and the odd response items was initially set to 31 degrees for the response item in the left hemifield and to 54 degrees for the response item in the right hemifield. After three right or three wrong responses, this difference value became smaller or larger respectively, with an initial step size of 6 degrees for the response item in the left hemifield or 11 degrees for the response item in the right hemifield. When a difference value of 13 degrees (left hemifield) or 21 degrees (right hemifield) had been reached, the step size was reduced to 2 degrees (left hemifield) or 3 degrees (right hemifield). The mean threshold of the reference group + 1.64**SD,* used as the fixed difference value for the fixed visual task, was 13 degrees for the response item in the left hemifield and 21 degrees for the response item in the right hemifield. The cut-off score for the fixed visual task (mean score of the HC group on this task minus 1.64*SD was 7.9 (left visual field) or 8.7 (right visual field). A score of patients below this cut-off was treated as deviant.

**Contrast perception.** Contrast perception was assessed with black (xyY 0, 0, 0 cd/m^2^) and grey (xyY = 0.265, 0.286, 0.191cd/m^2^) lines, in either a horizontal or a vertical orientation (length: 4º of visual angle). Subjects had to indicate which of the response items was similar to the target stimulus, i.e. in which of the odd response items did the lines have the same orientation as the target stimulus. For the adaptive task, the amount of contrast was manipulated by either decreasing or increasing the RGB-values of the grey lines. After three correct or incorrect responses, the RGB-values of the grey lines were decreased or increased respectively with 3 RGB (≈ xyY = 0.252, 0.266, 0.006 cd/m^2^). The starting value was RGB 16, 16, 16 (≈ xyY = 0.265, 0.286, 0.191cd/m^2^). When an RGB value of 7, 7, 7 (≈ xyY = 0.258, 0.276, 0.034cd/m^2^) had been reached, the step size was reduced to 1 RGB (≈ xyY = 0.258, 0.276, 0.034cd/m^2^). The fixed difference value for the fixed visual task (i.e. the mean threshold of the reference group + 1.64**SD*) was 7 RGB-steps. The cut-off score for the fixed visual task (mean score of the HC group on this task minus 1.64*SD was 9.03 (left visual field) or 9.48 (right visual field). A score of patients below this cut-off was treated as deviant.

**Glossiness perception.** The target stimulus and response items for the assessment of glossiness perception, were selected from 51 oval shaped figures (length: 4º of visual angle and width: 2º of visual angle) with a differing amount of glossiness, gradually changing from much glossiness (xyY = 0.297, 0.305, 4.741 cd/m^2^) to few glossiness (xyY = 0.290, 0.306, 6.521 cd/m^2^). For the adaptive task, the difference value between the target stimulus and the odd response item was initially set to 50 for both hemifields. After three right or three wrong responses, the difference value became smaller or larger respectively, with an initial step size of 5. When a difference value of 35 had been reached, the step size was reduced to 3. The fixed difference value for the fixed visual task (the mean threshold of reference group + 1.64**SD*) was 43 steps. The cut-off score for the fixed visual task (mean score of the HC group on this task minus 1.64*SD was 4.96 (left visual field) or 5.73 (right visual field). A score of patients below this cut-off was treated as deviant.

**Texture discrimination.** For the assessment of texture perception, 22 Brodatz texture pictures from the original Brodatz grayscale texture album (Brodatz, 1966) were used (size: squares with sides of 4º of visual angle). The Brodatz pictures are composed of 112 grayscale images of different grayscale textures, and are widely used as a validation dataset for texture classification and texture segmentation. The same paradigm as for the other tasks was employed, in which participants had to indicate which response item was similar to the target stimulus. However, in contrast to the other tasks this task did not contain any difference values. The 22 pictures could not be placed on a gradually changing spectrum, as it was not possible to tell whether a difference between e.g. picture 1 and 2 was similar to a difference between picture 5 and 6. The target item and the odd response item were pseudo-randomly chosen from a fixed distribution. This task consisted of 32 trials (16 per hemifield) and the raw score was the number of correct responses. The cut-off score for the visual task (mean score of the HC group minus 1.64*SD) was 11.2 (left visual field) or 11.6 (right visual field). A score of patients below this cut-off was treated as deviant.

**Motion perception.** The task assessing motion perception only involved a target stimulus, presented at 6º of visual angle on the left or right side of the red fixating dot. These target stimuli were circles (xyY = 0.285, 0.312, 23.766 cd/m^2^, diameter: 4.5º of visual angle) with 200 randomly arranged, moving dots in it (0.04º of visual angle each). All small dots either moved in a random direction, or they moved upwards or downwards. The percentage coherence of these moving dots (in relation to percentage moving dots in random fashion) was adaptively changed across trials and participants had to indicate whether the dots were moving upwards or downwards. The raw score was the threshold, which was the lowest percentage coherence of the moving dots on which at least three times in a row a correct response had been given. The starting value of the percentage coherence was initially set to 56 percent coherence. After three right or three wrong responses, the percentage coherence was reduced or increased respectively, with an initial step size of 8 percent. When a coherence percentage of 32 had been reached, the step size was reduced to 4 percent. Patients also performed this adaptive task. The cut-off score for this task was 38.4 (left visual field) or 34.6 (right visual field). A score of patients *above* this cut-off was treated as deviant.
